# Role function and job satisfaction of community nurses in China: A cross‐sectional study

**DOI:** 10.1002/nop2.2109

**Published:** 2024-01-31

**Authors:** Yuxin He, Yan Guo, Xiaoyuan Guo, Na Han, Bo Xin, Yixin Wu, Qiuyuan Wan, Panpan Shi, Shan Yang, Waleed Ksebe, Wenhui Jiang

**Affiliations:** ^1^ School of Nursing, Health Science Center Xi'an Jiaotong University Xi'an China; ^2^ School of Nursing Shaanxi University of Chinese Medicine Xianyang China; ^3^ Dongguan South Street Community Health Service Center Xi'an China

**Keywords:** China, community nurses, job satisfaction, role function

## Abstract

**Aim:**

To evaluate role function and job satisfaction, determine their relationship, and explore the factors influencing job satisfaction among community nurses in China.

**Design:**

Cross‐sectional study.

**Methods:**

This study was conducted between March and June 2020 on a cluster random sampling of 302 community nurses from 24 community health centres and stations in Xi'an, China. Self‐reported data were collected using the Demographics Questionnaire, Role Function of Community Nurses Questionnaire, and Job Satisfaction of Community Nurses Scale. Descriptive statistics, Pearson's correlation analysis, and multiple linear regression analyses were performed to analyse data.

**Results:**

Community nurses' main role function was organiser and manager (*M* = 2.56, SD = 0.987) and coordinator (*M* = 2.43, SD = 0.971). The lowest job satisfaction was for salary and benefits (*M* = 3.12, SD = 0.891) and personal development (*M* = 3.65, SD = 0.738). A positive correlation was found between the roles of caregiver, educator, navigator, and salary and benefits (*p* < 0.05) among community nurses. Multiple linear regression analyses indicated that monthly income and working experience in nursing explained 61.1% of the variance in job satisfaction.

## 1 INTRODUCTION

The need for community health services is increasing dramatically worldwide (Li et al., 2020). Community health centres (CHCs) are critical to the community healthcare system and have grown by 70% in the last decade (Martin et al., 2020). In China, approximately 10,122 CHCs and 26,038 community health stations (CHSs) were established in urban areas by 2021 (National Health and Family Planning Commission of the People's Republic of China, 2021). As a crucial component of community health services, community nursing is a continuous, dynamic, and all‐encompassing nursing professional service offered to locals with the ultimate objective of improving and sustaining the population's health (Xing et al., 2022). An increase in the number of community nurses has led to enhanced community nursing. It was reported that the number of community nurses in China would reach approximately 219,574 in 2020 (Ministry of Health of the People's Republic of China, 2020). However, there is a dearth of literature on the role function and job satisfaction of community nurses in China.

As prominent representatives of community nursing, community nurses' role function is strongly related to the quality of their work. In developed countries, community nurses have mastered multidisciplinary knowledge and skills and assumed their role function, including caregiver, educator, navigator, manager, coordinator, leader, and researcher (Buerhaus et al., 2015; Poghosyan et al., 2014). In China, community nurses provide a broad range of services, including illness prevention, healthcare, medical treatment, rehabilitation, health education, and guidance on family planning (Li et al., 2017). However, previous studies have reported that community nurses are still mainly caregiver and health educator, with less involvement in other roles, such as navigator, manager, and researcher (Li, 2012; Liu & Zhou, 2011a, 2011b). Therefore, in light of the community health system's remarkable progress, promoting the development of community health services requires further clarifying community nurses' multiple role function.

As an important predictor of nurses' intention to stay, job satisfaction has been related to positive emotional responses to working situations, meeting desired needs, and job values or equity (Niskala et al., 2020). A previous five‐country survey found that overall job satisfaction among community nurses was low, with high workload being the main cause of this finding (Dingley & Yoder, 2013). According to the Primary Care Workforce Survey, primary healthcare providers in China reported an overall job satisfaction rate of 47.6% (Shi et al., 2014). It has been discovered that severe burnout, low job satisfaction, and retention are common among community nurses (Li et al., 2014; Luo et al., 2021). Previous studies have also reported that the level of job satisfaction of community nurses varies greatly depending on the nature of their work (Ayalew et al., 2019; Tao et al., 2017). However, efforts to improve community nursing quality and build an integrated, cooperative community healthcare system are hampered by the lack of clarity regarding the factors influencing job satisfaction and its relationship with role function among community nurses.

In the context of the increasingly mature development of community health services in China, there are few relevant studies on whether community nurses' role function has been fully performed and to what extent their role function is associated with job satisfaction. Therefore, this study was conducted to evaluate role function and job satisfaction, determine their relationship, and explore the factors influencing job satisfaction among community nurses in China.

## 2 METHODS

### 2.1 Design

This cross‐sectional questionnaire‐based survey used a descriptive correlation research design to confirm community nurses' role function and job satisfaction in China.

### 2.2 Participants

Between the months of March and June 2020, a cluster random sampling method was employed in Xi'an, a city in western China. Out of the 150 CHCs and CHSs in Xi'an, a random selection of 24 facilities was made using the cluster method. Next, 302 valid samples were sent back among all nurses (320) in 24 CHCs and CHSs distributed anonymous self‐report questionnaires (response rate = 94%). The inclusion criteria for nurses included holding a nurse practitioner licence, being registered on duty, working full‐time in CHCs or CHSs for at least a year, and being open to participate in the study. Nurses with mental impairment were excluded.

A formula of cross‐sectional studies $n=\frac{\mu_{\alpha /2}^{2}/\sigma^{2}}{\delta^{2}}$ $n=\frac{\mu_{\alpha /2}^{2}/\sigma^{2}}{\delta^{2}}$ was adopted to estimate the sample size with an alpha level of 0.05 (Yan & Xu, 2015). The job satisfaction of community nurses in the pre‐test survey was 87.2% (*δ* = 3%), substituting into the formula to obtain *n* = 271. Assuming an 85% response rate, 320 community nurses were invited to participate in the study.

### 2.3 Measurements

#### 2.3.1 Demographic questionnaire

The self‐designed Demographic Questionnaire contained sociodemographic data, including age, gender, education level, monthly income, and length of working experience in nursing. Nurses were also asked about their professional positions, work shifts, employment, and previous training in community nursing.

#### 2.3.2 Role Function of Community Nurse Questionnaire

The Chinese version of the Role Function of the Community Nurse Questionnaire was modified by Liu and Zhou (2011b). Delphi method was adopted to finalise scale content. The questionnaire comprised six role functions of community nurses: caregiver, educator, navigator, organiser and manager, coordinator, observer, and researcher. Each item was scored on a 4‐point Likert scale ranging from 1 (“never or almost never”) to 4 (“always”), with a higher score signifying more role function participation. The survey's internal consistency was determined by Cronbach's α, resulting in a coefficient of 0.964. Additionally, split‐half reliability analysis yielded a coefficient of 0.634. Furthermore, the expert inquiry results indicated that the Scale of Content Validity Index (S‐CVI) attained a score of 0.970, exhibiting favourable reliability and validity.

#### 2.3.3 Job Satisfaction of Community Nurses Scale

A Chinese version of the Job Satisfaction of Community Nurses Scale was developed by Tao et al. (2009) and Ellenbecker et al. (2008). Delphi method was adopted to finalise scale content, which comprises 35 items in eight domains: salary and benefits (three items), management (four items), family/work balance (three items), workload (five items), relationship with colleagues (four items), personal development (five items), job content (four items), and work recognition (seven items). A higher score denotes more job satisfaction on a 5‐point Likert scale, which ranges from 1 (“absolutely disagree”) to 5 (“absolutely agree”). The survey's internal consistency was determined by Cronbach's α, resulting in a coefficient of 0.858. Additionally, split‐half reliability analysis yielded a coefficient of 0.718. Furthermore, the expert inquiry results indicated that the S‐CVI attained a score of 0.966, exhibiting favourable reliability and validity.

### 2.4 Data collection

After obtaining permission from the community health service department in charge of the Health Bureau in Xi'an, the participants were contacted and screened in the CHCs and CHSs. After obtaining informed consent, related questionnaires and scales were distributed to the participants. The researchers collected and double‐checked the questionnaires and scales after the participants completed them.

### 2.5 Reliability evaluation

To evaluate reliability, we used the procedure corrections suggested by Podsakoff et al. (2011). We segmented questionnaires such that responders had to pause and carefully read instructions, which helped psychologically separate the measures. To reduce item ambiguity, we kept items specific. Moreover, we kept the questionnaires as brief as possible to retain the incentive to respond accurately. We also guaranteed anonymity to reduce the tendency to respond in a socially desirable manner.

### 2.6 Statistical analyses

EpiData 3.0 was used to enter the data, and SPSS 22.0 was applied for the analysis. Demographics are presented as *N* (%). The mean and standard deviation were calculated for each item of the questionnaire on role function and job satisfaction. Considering that these subscales were ratio scale of measurement, a Pearson correlation analysis was performed to determine the relationship between role function and job satisfaction of community nurses, T‐test and ANOVA were used to preliminarily analyse the factors influencing job satisfaction, and multiple linear regression with a stepwise approach was used to determine the factors influencing job satisfaction. The significance level was set at *p* < 0.05.

### 2.7 Ethical considerations

This study was approved by the Ethics Board of Xi’an Jiaotong University (Reference No.2018‐515). All participants in this study were voluntary, and informed consent was obtained. The anonymity and confidentiality of the responses were protected in this study.

## 3 RESULTS

### 3.1 Participants' characteristics

A total of 302 qualified questionnaires and scales from 24 community health service centres were returned, with a response rate of 94%. The demographic characteristics of the participants are summarised in Table 1. The majority were female (96.0%), married (75.2%), younger than 45 years of age (86.0%), had received an associate degree (64.9%), and held a junior position (67.8%). A total of 61 (22.2%) claimed to have entered the field of community nursing without previous training, and 175 (58%) claimed to have fewer than 10 years of working experience in nursing.

**TABLE 1:** Demographic details of community nurses (*n* = 302).

Variable	Categories	Number of patients	*N* (%)
Age (years)	<25	59	19.5
	25–34	132	43.7
	35–45	69	22.8
	>45	42	13.9
Gender	Male	12	4.0
	Female	290	96.0
Education level	High school	2	0.7
	Diploma	54	17.9
	Associate degree	196	64.9
	Bachelor degree	50	16.6
Professional position	Junior	200	66.2
	Middle	94	31.1
	Senior	8	2.7
Working experience in nursing (years)	<5	95	31.5
	5–10	80	26.5
	>10	127	42.1
Work shifts	Yes	90	29.8
	No	212	70.2
Previous training in community nursing	Yes	241	79.8
	No	61	20.2
Monthly income, RMB	<3000	24	7.9
	3001–5000	216	71.5
	>5000	62	20.5
Employment	Permanent	103	34.1
	Contract	166	55.0
	Others	33	10.9

### 3.2 Role function of community nurses

The scores for each role function from the highest to the lowest were organiser and manager (*M* = 2.56, SD = 0.987), coordinator (*M* = 2.43, SD = 0.971), educator (*M* = 2.38, SD = 0.840), caregiver (*M* = 2.34, SD = 0.868), observer and researcher (*M* = 2.29, SD = 0.997), and navigator (*M* = 2.09, SD = 0.987), as shown in Table 2.

**TABLE 2:** The role function of community nurses (*n* = 302).

Items	Minimal values	Maximum values	*M* (SD)
Organiser and manager	1.00	4.00	2.56 (0.987)
Coordinator	1.00	4.00	2.43 (0.971)
Educator	1.00	4.00	2.38 (0.840)
Caregiver	1.00	4.00	2.34 (0.868)
Observer and researcher	1.00	4.00	2.29 (0.997)
Navigator	1.00	4.00	2.09 (0.987)

Abbreviations: M, mean; SD, standard deviation.

### 3.3 Job satisfaction of community nurses

The scores for each dimension of job satisfaction from lowest to highest were salary and benefits (*M* = 3.12, SD = 0.891), personal development (*M* = 3.65, SD = 0.738), work content (*M* = 3.67, SD = 0.645), workload (*M* = 3.79, SD = 0.623), management (*M* = 4.20, SD = 0.752), family/work balance (*M* = 4.26, SD = 0.701), job recognition (*M* = 4.41, SD = 0.664), and relationship with colleagues (*M* = 4.56, SD = 0.594), as shown in Table 3.

**TABLE 3:** Job satisfaction of community nurses (*n* = 302).

Items	Minimal values	Maximum values	*M* (SD)
Relationship with colleagues	2.25	5.00	4.56 (0.594)
Work recognition	1.00	5.00	4.41 (0.664)
Family/work balance	1.67	5.00	4.26 (0.701)
Management	1.00	5.00	4.20 (0.752)
Workload	1.80	5.00	3.79 (0.623)
Job content	1.50	5.00	3.67 (0.645)
Personal development	1.60	5.00	3.65 (0.738)
Salary and benefits	1.00	5.00	3.12 (0.891)

Abbreviations: M, mean; SD, standard deviation.

### 3.4 Correlation between role function and job satisfaction

Pearson's correlation analysis was conducted to investigate the relationship between role function and job satisfaction. Community nurses' role function (*r* = 0.153, *p* < 0.01) and its dimensions of caregiver (*r* = 0.147, *p* < 0.05), educator (*r* = 0.117, *p* < 0.05), navigator (*r* = 0.166, *p* < 0.01), organiser and manager (*r* = 0.153, *p* < 0.01), and observer and researcher (*r* = 0.132, *p* < 0.05) were positively associated with the salary and benefits dimension of job satisfaction; organiser and manager (*r* = 0.134, *p* < 0.05) and coordinator (*r* = 0.134, *p* < 0.05) were positively correlated with the management dimension, as shown in Table 4.

**TABLE 4:** The Pearson correlation analysis between the role function and job satisfaction of community nurses (*n* = 302).

	Total score	Caregiver	Educator	Navigator	Organiser and manager	Coordinator	Observer and researcher
Total score	0.070	0.046	−0.010	0.092	0.116	0.112	0.058
Salary and benefits	0.153**	0.147*	0.117*	0.166**	0.153**	0.112	0.132*
Management	0.092	0.071	0.027	0.087	0.134*	0.134*	0.060
Workload	−0.001	0.217	−0.088	0.022	0.079	0.037	0.008
Family/work balance	0.014	0.007	−0.047	0.041	0.057	0.051	0.002
Relationship with colleagues	0.006	−0.020	−0.074	0.017	0.054	0.077	0.024
Personal development	0.069	0.026	0.000	0.105	0.101	0.110	0.060
Job content	−0.035	−0.012	−0.068	−0.016	−0.026	0.025	−0.057
Work recognition	0.101	0.071	0.027	0.114*	0.136*	0.129*	0.092

*p < 0.05, **p < 0.001, denoted statistical significance.

### 3.5 Factors influencing the job satisfaction of community nurses

The factors influencing community nurses' job satisfaction were age (*F* = 5.993, *p* < 0.01), education level (*F* = 19.009, *p* < 0.01), professional position (*F* = 5.722, *p* < 0.01), working experience of nursing (*F* = 8.690, *p* < 0.01), monthly income (*F* = 229.865, *p* < 0.01), and employment (*F* = 13.461, *p* < 0.01). Other demographic variables, including gender, work shifts, and previous training in community nursing, did not significantly affect job satisfaction, as shown in Table 5. Multiple regression analyses were performed to investigate the factors influencing job satisfaction based on univariate analysis of community nurses' job satisfaction scores. The results showed that monthly income (*β* = 0.765, *p* < 0.01) and working experience in nursing (*β* = −0.097, *p* < 0.01) were the main factors influencing community nurses' job satisfaction. The cumulative effect of monthly income and working nursing experience on community nurses' job satisfaction was 61.1%, as shown in Table 6.

**TABLE 5:** Influencing factors of job satisfaction among community nurses (*n* = 302).

Variable	Categories	Job satisfaction	*F*/*t*	*p*
Age (years)	<25	139.58 ± 19.01	5.993	0.001
	25–34	144.50 ± 17.36		
	35–45	136.10 ± 19.62		
	>45	132.02 ± 21.78		
Gender	Male	139.83 ± 18.31	−0.010	0.992
	Female	139.88 ± 19.40		
Education level	High school	136.50 ± 19.09	8.955	< 0.001
	Diploma	134.59 ± 19.80		
	Associate degree	143.85 ± 17.00		
	Bachelor degree	130.20 ± 22.88		
Professional position	Junior	142.74 ± 18.36	5.722	0.001
	Middle	135.12 ± 20.37		
	Senior	118.60 ± 4.83		
Working experience in nursing (years)	<5	134.00 ± 16.52	8.690	< 0.001
	5–10	143.04 ± 17.91		
	>10	144.50 ± 16.02		
Work shifts	Yes	134.61 ± 21.06	0.771	0.442
	No	141.18 ± 18.74		
Previous training in community nursing	Yes	139.50 ± 19.94	−0.764	0.447
	No	141.41 ± 16.78		
Monthly income, RMB	<3000	118.33 ± 12.48	229.865	< 0.001
	3001–5000	149.35 ± 11.54		
	>5000	115.24 ± 14.10		
Employment	Permanent	132.22 ± 20.49	13.461	< 0.001
	Contract	144.19 ± 17.51		
	Others	142.12 ± 17.49		

**TABLE 6:** Multiple regression analysis for the influencing factors of job satisfaction among community nurses (*n* = 302).

	*B*	*β*	*R*	Adjust *R* ^2^	*t* value	*p* Value
Fixed effect	121.133		0.783	0.611	53.120	< 0.001
Monthly income	32.713	0.765			21.108	< 0.001
Working experience in nursing, years	−2.206	−0.097			−2.684	0.008

*Note*: Dependent variable: job satisfaction, 95% confidence level (*α* = 0.05).

Abbreviations: *B*, unstandardised regression coefficients; *β*, standardised regression coefficients; *R*, correlation coefficients; *R* ^2^, determination coefficient.

## 4 DISCUSSION

To the best of our knowledge, few studies have been undertaken to verify community nurses' role function and job satisfaction. Our study provides a new view of current community nurses' role function, job satisfaction, and the analysis of factors influencing job satisfaction in China. These findings suggest that the role function of community nurses has not been fully developed, and their overall job satisfaction needs to be improved. There was a positive correlation between role function and job satisfaction. The main factors affecting job satisfaction were monthly income and nursing working experience. These results provide information that can assist nursing managers in enhancing community nurses' role function and job satisfaction in their organisations.

In the present study, it was concluded that the age of community nurses in Xi'an is mainly 25–34 years old (43.7%), the educational level is mainly associate degree (64.9%), and the professional position is still mainly junior and middle (97.3%). This indicates that the overall level of academic qualifications and professional position of community nurses in Xi'an was low. Some of them still had not received previous training in community nursing, which is similar to the results of Yu et al. (2021). Community nursing in China began late, and its foundation was weak. The quantity and quality of community nurses have been an important reason for hindering the development of community nursing and their ability to fulfil all role function. Introducing professional talent and improving the training mode are urgent problems to solve.

Our results indicate that the role function of public health services in the work of community nurses is improving. Community nurses' most frequent role function was organiser and manager, such as organising community health checkups for the elderly and the chronically ill. However, we discovered that community nurses appear to fill the roles of navigator, observer, and researcher infrequently and overlook several tasks related to these roles, which is similar to previous findings (Ma et al., 2019; Yu et al., 2021). Community nurses in China are not yet performing their role function sufficiently, which may be related to two aspects: either they lack the ability to do so or they have not yet been given this responsibility (Phelan et al., 2018; Tao et al., 2019). The former must be accomplished by raising community nurses' professional proficiency and general calibre (Dupin et al., 2020). The latter calls for managers to strengthen and define the duties and obligations of community nurses, and their competency standards for them (O'Grady & Clavelle, 2021).

Regarding community nurses' job satisfaction, our findings indicate that overall job satisfaction must be improved. The higher scoring dimensions were relationship with colleagues, work recognition, and family/work balance. Salary and benefits, personal development, and job content received lower scores, consistent with comparable studies' outcomes (Lin et al., 2020; Ren et al., 2017). This may be attributed to community nurses being more proactive in making career decisions and the recent shift in national policy in favour of expanding community health services (Xu et al., 2022). Comparing surveys abroad, community nurses consider that salary and benefits are the most important aspects that need improvement (Barrett et al., 2016; McCreary, 2020), which is related to the overall shortage and uneven distribution of health resources in China (Zhang et al., 2015).

Investigating the relationship between role function and job satisfaction of community nurses revealed that the higher the satisfaction of community nurses with their salary and benefits, the better they assumed the roles of caregiver, educator, navigator, organiser and manager, and observer and researcher; similarly, the higher the satisfaction of community nurses with management, the better they performed the roles of organiser and manager, and coordinator. These findings provide inputs for community nursing managers to focus on the assumption of the nurse's role as navigator and improve their capacity to assume the role of navigator by improving the professional and work‐related aspects of nursing, work environment and resources, and patient care (Tao et al., 2017). Meanwhile, the job satisfaction of community nurses should be improved by increasing their salary and benefits, improving management to consolidate their original role function, and providing conditions for their expansion into new roles (Huy et al., 2018; Pu et al., 2019). It is worth noting that the correlation coefficient (r) obtained in our analysis did not reach a high level. We speculate that this could be attributed to certain limitations in our sampling methods, sample size, and geographical coverage, leading to a relatively weaker representation of the sample in relation to the overall population. Consequently, these factors have influenced the obtained results.

Another finding of our study was that age, education level, professional position, working experience in nursing, monthly income, and employment all significantly influenced job satisfaction. The results of the multiple regression analysis showed that monthly income and working experience in nursing were the main factors influencing the job satisfaction of community nurses. The highest‐paid community nurses were found to be the ones with the most satisfying job. Monthly income is the foundation of material security for individuals and families. The higher the monthly income, the more material requirements can be addressed and the better the satisfaction (Park & Han, 2013; Schuiling et al., 2019). Additionally, the results showed that community nurses with 5–10 years of experience had the highest job satisfaction, followed by those with fewer than 5 years of experience, while those with more than 10 years of experience had the lowest. Community nurses with 5–10 years of service have accumulated learning ability and working experience, are in a time of stability in both work and family, and can better adapt to the continuous policy changes and work requirements of community health services (Ashley et al., 2018; Tourangeau et al., 2015). Contrary to the findings of Ren et al. (2017), community nurses with more than 10 years of working experience had the lowest job satisfaction. This may be related to the nature of the work, salary distribution, and family pressure. Most nurses at this stage are in leadership positions and not only have to be competent in clinical work but also face complex management requirements, while the level of income is not proportional to the value of labour. These findings suggest that community nursing managers should strengthen the logistical support system and focus on the reasonableness and fairness of compensation and the current job status of low‐ and high‐ranking community nurses to improve job satisfaction (Chamanga et al., 2020).

## 5 LIMITATIONS

This study encompasses several limitations that should be taken into account. Firstly, the recruitment of participants was limited to the Western Chinese metropolis of Xi'an and the small sample size employed might restrict the generalisability of our findings. Future studies should include community nurses from diverse geographical regions and expand sample size. Furthermore, the cluster sampling method employed in this study did not adequately account for the variations across different groups, leading to a limited sample distribution and statistically significant sampling errors. Future research should consider employing standardised sampling methods and reporting data aggregation appropriately. Moreover, it is important to acknowledge that the utilisation of self‐reported measurements in our study might have affected the accuracy of the responses. The participants' mood and potential response bias could have influenced the outcomes to some extent. Hence, it is recommended that future research incorporates multiple measurements to comprehensively evaluate the associated variables.

## 6 CONCLUSION

Our findings indicate that the role function of community nurses is not fully functional, and job satisfaction still needs to be improved. There was a positive correlation between some aspects of role function and job satisfaction. Monthly income and working experience in nursing were the main predictors of community nurses' job satisfaction. Our findings suggest that nurse administrators should consider expanding community nurses' role function to facilitate job satisfaction, especially as researchers and navigators. Furthermore, promoting personal development and improving salary and benefits are necessary to enhance community nurses' job satisfaction. These findings emphasise the necessity of improving the role function of community nurses and highlight the importance of promoting personal development and improving salary and benefits is necessary in promoting nurses' job satisfaction. A future job satisfaction promotion programme for community nurses should consider these factors.

## AUTHOR CONTRIBUTIONS

YH, YG, and WJ contributed to the conception of the study; YH, YG, XG, NH, BX, YW, QW, PS, SY, WK, and WJ implemented the study; YH and YG performed the data analyses and wrote the manuscript; XG, NH, BX, YW, QW, PS, SY, WK, and WJ helped perform the analysis with constructive discussions; All authors read and approved the final manuscript as submitted.

## FUNDING INFORMATION

The study was supported by the Shaanxi Provincial Key Research and Development Program (2021SF‐190) and National Social Science Fund of China (22BGL253).

## CONFLICT OF INTEREST STATEMENT

No conflict of interest has been declared by the authors.

## ETHICS STATEMENT

This study was approved by the Ethics Board of Xi'an Jiaotong University (Reference NO.2018–515). The participants in this study were voluntary and the informed consent was obtained. The anonymity and confidentiality of the responses were protected in the study.

## Data Availability

The data that support the findings of this study are available from the corresponding author upon reasonable request. The data are not publicly available due to privacy or ethical restrictions.
